# An Integrated ERAS–PBM Pathway for Cardiac Surgery: Insights and Outcomes

**DOI:** 10.3390/jcm15218447

**Published:** 2026-10-30

**Authors:** Luc Lavanchy, Felix Rohrer, Zied Ltaief, Alexandra Othenin-Girard, Jerome Graf, Anna Nowacka, Ziyad Gunga, Mario Verdugo-Marchese, Valentine Melly, Matthias Kirsch, Carlo Marcucci, Valentina Rancati

**Affiliations:** 1 Department of Anesthesiology, Centre Hospitalier Universitaire Vaudois (CHUV), 1011 Lausanne, Switzerland; 2 Faculty of Biology and Medicine, University of Lausanne, 1011 Lausanne, Switzerlandjerome.graf@chuv.ch (J.G.);; 3 Department of Intensive Care Medicine, Centre Hospitalier Universitaire Vaudois (CHUV), 1011 Lausanne, Switzerland; 4 Department of Cardiovascular Surgery, Centre Hospitalier Universitaire Vaudois (CHUV), 1011 Lausanne, Switzerland

**Keywords:** ERAS, patient blood management, anemia, cardiac surgery, transfusion, perioperative care, outcomes

## Abstract

**Background/Objectives**: Anemia is a common condition in cardiac surgery and is strongly associated with adverse outcomes, including higher transfusion requirements, increased morbidity, and prolonged hospital stay. Enhanced Recovery After Surgery (ERAS) and Patient Blood Management (PBM) are both multimodal strategies designed to improve perioperative care. This study evaluates the impact of integrating ERAS and PBM with preoperative anemia management into a single standardized pathway in elective cardiac surgery. **Methods**: We conducted a retrospective, single-center before–after study at Lausanne University Hospital, comparing a pre-ERAS cohort (2019) with a prospective cohort managed under the ERAS–PBM pathway (May 2023–November 2024). The ERAS–PBM pathway included systematic preoperative anemia screening and treatment, reinforcement of adherence to existing restrictive transfusion thresholds, optimized blood conservation techniques, and standardized perioperative care bundles coordinated by a dedicated ERAS nurse. The primary outcome was the rate of red blood cell (RBC) transfusions during hospitalization. Secondary outcomes included overall transfusion rates, hemoglobin levels, complications, and length of stay. **Results**: A total of 391 patients were included (ERAS, *n* = 229; pre-ERAS, *n* = 162). RBC transfusion rates were significantly lower in the ERAS group (23.6% vs. 38.9%, *p* = 0.0012), with a reduction in the number of units per patient (*p* = 0.0005). Hemoglobin at admission was higher in the ERAS cohort (139.5 vs. 134 g/L, *p* = 0.0009). Overall complication rates decreased (46.3% vs. 66.7%, *p* < 0.0001), including major adverse cardiovascular events and pulmonary complications. Hospital stay was shorter (9 vs. 12 days, *p* < 0.0001), as was mechanical ventilation time (0 vs. 2.1 h, *p* < 0.0001). Anemic patients at admission were more transfused (39.3% vs. 18.6%, *p* = 0.002). Within the ERAS group, RBC transfusion was associated with higher rates of selected postoperative complications and longer ICU and hospital stays. In an exploratory multivariable logistic regression analysis, ERAS–PBM implementation was associated with lower odds of RBC transfusion during hospitalization (adjusted OR 0.59, 95% CI 0.36–0.98; *p* = 0.041). **Conclusions**: The integration of ERAS and PBM into a single multimodal pathway was associated with significant reductions in transfusion rates, complications, and length of stay in elective cardiac surgery. Anemia at admission and perioperative transfusion were associated with worse outcomes, emphasizing the importance of anemia management. Larger, multicenter prospective studies are warranted to confirm these results and to optimize anemia correction strategies, including earlier treatment of iron deficiency and outpatient erythropoietin use.

## 1. Introduction

Perioperative management in cardiac surgery remains a significant challenge. For over two decades, the Enhanced Recovery After Surgery (ERAS) Society has advocated for multimodal perioperative care pathways designed to minimize postoperative complications, reduce hospital length of stay, and facilitate a quicker return to normal activities. In 2019, ERAS recommendations for cardiac surgery were published, which included a bundle related to a Patient Blood Management (PBM) program, stipulating that if time permits, all causes of anemia should be investigated prior to surgery [1].

Indeed, anemia is a prevalent condition strongly associated with poorer outcomes in cardiac surgery [2,3]. A narrative review summarized 44 observational studies demonstrating that anemia is linked to increased morbidity, increased hospital length of stay (LOS), higher transfusion requirements, and even increased mortality [4]. These findings were corroborated by a large multicentre cohort study in the Netherlands involving 35,000 patients undergoing cardiac surgery, where anemia was present in 20% of patients [5]. Given this high prevalence and its association with worse perioperative outcomes, anemia represents a relevant clinical problem in cardiac surgical patients.

Preoperative anemia screening and treatment is included in the first pillar of PBM, a multimodal, patient-centred approach aimed at improving patient outcomes through management of preoperative anemia (pillar 1), reducing blood loss (pillar 2), and optimizing tolerance of anemia (pillar 3) [6]. However, PBM program implementation remains challenging and represents a major shift in clinical practice [7]. Barriers to implementation range from medical misconceptions regarding transfusion thresholds to logistical issues in administering iron therapy in a timely manner, as well as reimbursement difficulties.

Our cardiac surgery ERAS program at the Lausanne University Hospital (CHUV) has been ongoing since May 2023 and achieved ERAS Society certification in October 2023. Our roadmap for implementation was previously published [8]. Pre-implementation analysis revealed that preoperative anemia management was inconsistent, and there was a lack of formal strategies to address this issue. Therefore, we combined our ERAS pathway with multimodal PBM measures, as described by Meybohm et al. [4].

The PBM and cardiac ERAS programs were initially developed by two independent teams. During the development of the cardiac ERAS program, the existing PBM team was involved in the project, enabling PBM principles and expertise to be integrated into a single ERAS–PBM pathway for elective cardiac surgery.

This article details the implementation of two multimodal concepts—ERAS and PBM—in elective cardiac surgery and presents the initial results from the first 18 months. The primary objective is to evaluate the impact on postoperative outcomes, particularly in terms of reducing blood transfusions and improving patient recovery.

## 2. Materials and Methods

This was a single-centre, retrospective observational study that compared two patient cohorts undergoing elective cardiac surgery at the Lausanne University Hospital (CHUV): one prospective cohort managed under the cardiac ERAS–PBM pathway (ERAS group), and a historical cohort from 2019 prior to ERAS implementation (pre-ERAS group), selected as a reference because it represents the last period of activity unaffected by the COVID-19 pandemic.

The study was carried out in alignment with the principles of the Declaration of Helsinki and was approved by the Ethics Committee of the Canton of Vaud for research involving human subjects (CER-VD #2024-00632). The study complies with the STROBE (Strengthening the Reporting of Observational Studies in Epidemiology) guidelines for observational research reporting. Data for the ERAS group were obtained from the cardiac ERAS program’s REDCap (Research Electronic Data Capture; Vanderbilt University, Nashville, TN, USA; version 17.3.10) database [9]. All variables for data collection were specified prior to program initiation. During the ERAS implementation phase, patients were prospectively and consecutively recruited, thereby establishing a prospective cohort of ERAS patients. For the retrospective cohort, data were extracted from the hospital’s electronic medical records and subsequently entered into the same REDCap database.

### 2.1. Objective

The primary objective of this study was to evaluate the impact of the multimodal ERAS–PBM pathway on postoperative outcomes, particularly focusing on transfusion rates and patient recovery.

The primary outcome was the rate of red blood cell (RBC) transfusions during the hospital stay.

Secondary outcomes included a range of measures related to transfusion and recovery. Notably, RBC consumption was assessed at different time points: intraoperatively, within the first 12 h after surgery, and after 12 h post-surgery. We also calculated the transfusion index, defined as the total number of blood units transfused divided by the number of patients who received a transfusion. Other secondary outcomes were transfusion rates of other blood products such as fresh frozen plasma (FFP) and platelets, and of the factor concentrates prothrombin complex concentrate (PCC) and fibrinogen, as well as the hemoglobin value on the day before surgery. We evaluated the length of hospital and ICU stay, and the duration of mechanical ventilation. We monitored the rate of major adverse cardiovascular events (MACE) which included myocardial infarction, stroke, acute heart failure, cardiac arrest, and death. We also monitored the rate of pulmonary complications (pneumonia, pleural effusion, respiratory failure, pneumothorax), infectious complications (pneumonia, wound infection, urinary infection, mediastinitis, sternotomy infection, vascular access or vein graft infection, sepsis, and septic shock), renal complications (including renal failure and the need for transient renal replacement therapy), and thrombotic events (pulmonary embolism and deep venous thrombosis).

Additionally, we evaluated the rate of compliance with anemia screening and treatment protocols (pillar 1 of PBM) as a secondary outcome.

### 2.2. ERAS–PBM Pathway

Within the framework of our ERAS–PBM pathway, eight overarching objectives have been defined and implemented throughout the patient journey:

1. Preoperative patient education: consultation with an ERAS nurse to assess tobacco and alcohol consumption and provide education on home-based prehabilitation.
2. Optimization of nutrition and glycemic control: screening for malnutrition, optimizing nutritional intake, and ensuring adequate glycemic control preoperatively. Postoperative nutritional intake and glycemic management are closely monitored.
3. Patient Blood Management: preoperative screening and correction of anemia, timely discontinuation of antithrombotic drugs, reduction in intraoperative blood loss, and minimization of postoperative transfusions.
4. Infection-prevention care bundle: including preoperative hygiene, nasal mupirocin decolonization, antibiotic prophylaxis, sternal fixation strategies, and appropriate wound dressing.
5. Reduction in invasive mechanical ventilation duration: through patient-tailored combined anesthesia and analgesia, depth of anesthesia monitoring and optimized temperature management to support faster respiratory recovery.
6. Hemodynamic control: goal-directed fluid therapy, monitoring by echocardiography, prevention and management of postoperative atrial fibrillation.
7. Pain management and opioid side effect mitigation: multimodal analgesia strategies and chest wall blocks with reduced opioid consumption to decrease the incidence of opioid-related side effects.
8. Early mobilization and delirium prevention.

The ERAS certification process itself was recently published by Ltaief et al. [8]. To obtain the certification, the ERAS Society (Stockholm, Sweden) mandates a comprehensive system featuring well-defined, widely recognized protocols and adherence rates above 70%. Several components of PBM were already in use at our institution before the implementation of ERAS, such as the routine use of tranexamic acid during surgery, the systematic use of cell salvage, the monitoring and correction of coagulopathy with point-of-care viscoelastic testing (ROTEM^®^ sigma; Tem Innovations GmbH, Munich, Germany) and the intraoperative application of a restrictive transfusion protocol [7]. Other measures, however, were not routinely practiced, including systematic screening and correction of anemia and consistent use of retrograde priming during CPB. The restrictive RBC transfusion protocol itself remained unchanged between the pre-ERAS and ERAS–PBM periods. It specified a standard hemoglobin threshold of 70 g/L. Between 70 and 90 g/L, transfusion could be considered in the presence of signs of inadequate oxygen delivery (dyspnea, electrocardiographic signs of ischemia, lactic acidosis, low mixed venous oxygen saturation, tachycardia and/or hypotension) or clinically active coronary artery disease, heart failure, or cerebrovascular disease. RBC transfusion was not indicated at hemoglobin levels above 90 g/L, irrespective of the clinical context. The ERAS–PBM pathway included specific staff education to reinforce its consistent application.

The preoperative anemia screening and treatment (first pillar of PBM) was included in the ERAS–PBM pathway and is based on several key principles: timely screening and diagnostic work-up, correction of iron deficiency anemia prior to hospital admission, and a short-term boost with erythropoietin at admission in cases of persistent anemia. This process is coordinated by the dedicated ERAS nurse directly with the patient.

Initial anemia screening was ideally performed approximately six weeks before the scheduled surgery, or as early as possible when this timeline cannot be met. To this effect, the preoperative pathway was adapted, and patients were scheduled earlier for the surgical and anesthetic consultations. Prior to the surgical consultation, a letter was sent to the patient requesting preoperative anemia screening be performed by the primary care physician. However, this was often not completed. Therefore, after confirmation of the surgical indication, the patient was scheduled for a prehospital assessment, including the anesthetic consultation, a consultation with the ERAS nurse, and blood sampling for anemia and iron deficiency screening. The results of the work-up were reviewed directly by the trained ERAS nurse, who initiated substitution therapy according to the predefined algorithm. When questions arose, the ERAS nurse discussed the case with either the cardiologist affiliated with the Department of Cardiac Surgery, the hospital’s PBM coordinator, or a consultant from the cardiac anesthesia team. Treatment was then scheduled either with the primary care physicians or in the hospital, depending on patient preference. In most cases, iron supplementation could be coordinated directly by the ERAS nurse with the primary care physician.

**Figure 1:**
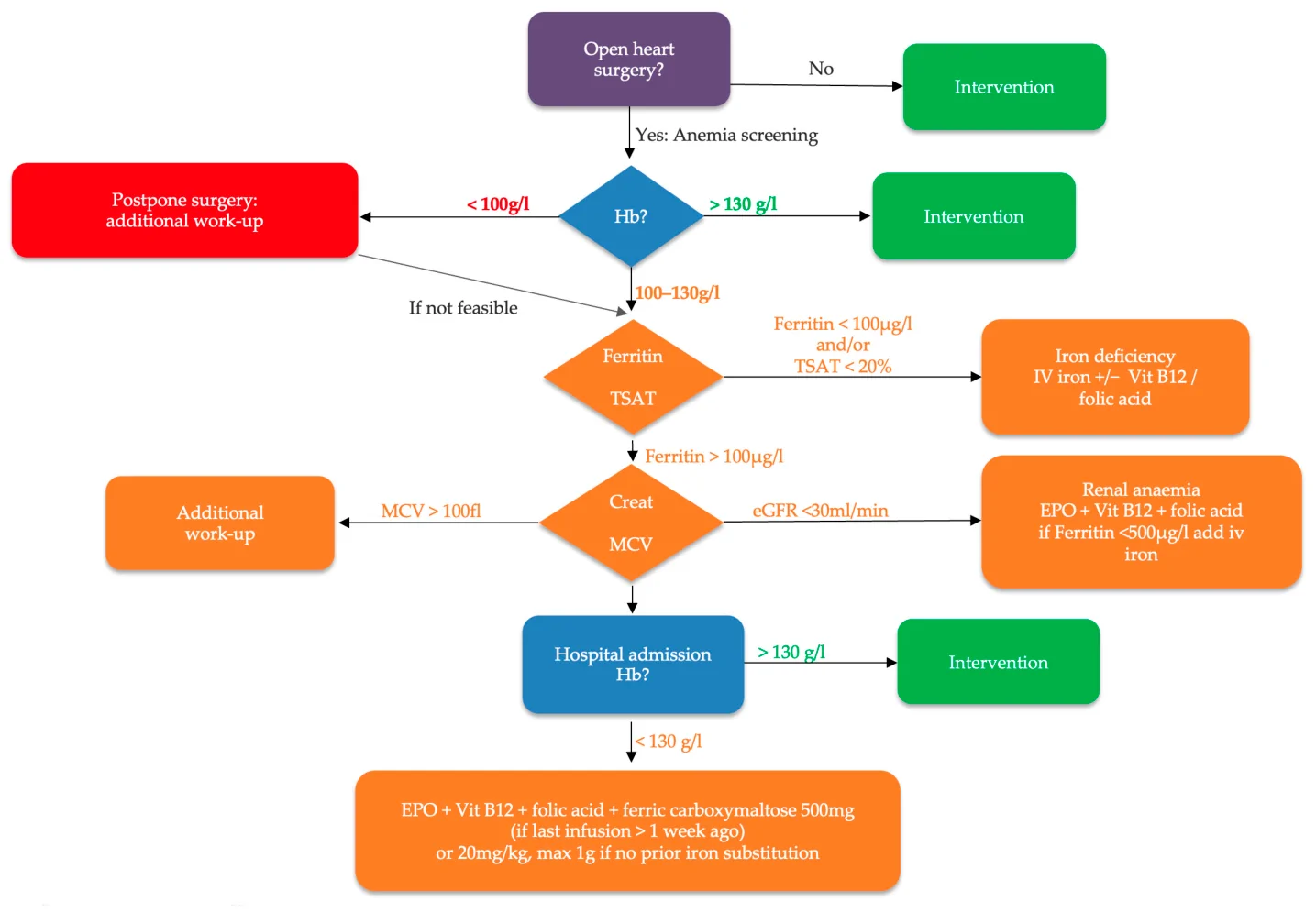
Preoperative anemia treatment algorithm Hb: hemoglobin, TSAT: transferrin saturation, creat: creatinine, MCV: mean corpuscular volume, eGFR: estimated glomerular filtration rate, EPOα: erythropoietin alpha.

The initial anemia work-up included a full blood count, ferritin, transferrin saturation coefficient (TSAT), and serum creatinine. Anemia was defined as a hemoglobin level of <130 g/L for both genders, in accordance with PBM guidelines. When anemia was present, it was categorized by etiology into four groups: iron deficiency anemia, renal anemia, vitamin B12 or folate deficiency, and anemia of inflammation [10]. The treatment algorithm is illustrated in Figure 1.

For treatment, iron deficiency was managed with intravenous iron supplementation, most often ferric carboxymaltose (20 mg/kg, max. 1 g). In patients with renal impairment, erythropoiesis-stimulating agents were added as part of renal anemia management. Folic acid (5 mg orally daily) and vitamin B12 (1 mg subcutaneously) were routinely co-administered with iron to support erythropoiesis, or at higher doses in cases of confirmed deficiency. In the case of anemia of inflammation, treatment (erythropoietin α, IV iron, vitamin B12, and folic acid) was administered only upon hospital admission. The dosing consisted of a single dose of 40,000 IU erythropoietin alpha (EPOα).

At hospital admission, one day before surgery, hemoglobin levels were rechecked. In cases of persistent anemia, a boost with erythropoietin and completion of the total iron dose according to the Ganzoni formula [11] were prescribed by the surgical resident. At this stage, the intervention is no longer delayed, even in cases of persistent anemia.

To support the implementation of the ERAS–PBM pathway, we launched both external and internal educational initiatives. Externally, an information form was provided to general practitioners. Internally, three structured training cycles were conducted for anesthesiologists, surgeons, intensivists, and cardiologists managing postoperative care on the ward.

### 2.3. Participants

The ERAS–PBM program was implemented in May 2023 as a clinical quality-improvement initiative. The ERAS–PBM cohort comprises elective patients managed within the clinical program between May 2023 and November 2024. The control group consists of patients who met the inclusion criteria during 2019, the last year in which the cardiac surgery program was unaffected by the COVID-19 pandemic. Inclusion criteria were patients aged 18 years or older who underwent elective cardiac surgery via median sternotomy with cardiopulmonary bypass, regardless of the surgical complexity or history of prior cardiac surgery. Exclusion criteria were patients undergoing urgent surgeries, ventricular assist device implantation, heart transplantation, or those who declined to provide general consent for data reuse.

### 2.4. Variables and Data Collection

The variables collected for clinical quality monitoring were predefined before implementation of the ERAS–PBM program. Clinical data were prospectively and systematically recorded in the dedicated ERAS REDCap database for quality monitoring. For the purposes of the present research study, these routinely collected data were subsequently retrieved and analyzed following ethics approval. For the retrospective cohort, data were extracted from the hospital’s electronic medical records and subsequently entered into the same database. The same variable definitions were applied to both cohorts whenever the corresponding information was available in the electronic medical records.

Baseline patient characteristics included: age, gender, body mass index (BMI), smoking status, alcohol consumption, recreational drug use, arterial hypertension, diabetes mellitus, dyslipidemia, chronic pulmonary disease, preoperative serum creatinine, peripheral arterial disease (PAD), cerebrovascular disease, American Society of Anesthesiologists (ASA) physical status classification, and left ventricular ejection fraction (LVEF).

Surgical characteristics collected comprised: type of surgery, specific procedure performed, aortic cross-clamp time, cardiopulmonary bypass (CPB) duration, redo surgery, use of isolated cerebral perfusion, and systemic circulatory arrest.

Regarding haemoglobin values in the ERAS group, two different preoperative values were available: pre-admission (Pre-admission Hb) during ambulatory consultation and control at admission (Admission Hb). For most cases in the pre-ERAS group, only the Admission Hb value was available, as there was no pre-admission screening for anemia.

The other data included patient follow-up monitoring data, as well as data used for monitoring the quality of the ERAS protocol.

EuroSCORE II was retrospectively calculated, based on available preoperative clinical data.

### 2.5. Statistical Analysis

Comparisons between the pre-ERAS and ERAS groups were conducted for transfusion rates, complication rates, length of stay, hemoglobin levels, and the number of blood products transfused. Normality of continuous variables was assessed using the Shapiro–Wilk test. As all continuous variables showed non-normal distributions, they are presented as median [interquartile range (IQR)]. Categorical variables are presented as absolute numbers and percentages.

Categorical variables were compared using Pearson’s chi-square test or Fisher’s exact test when expected frequencies were below 5. All statistical tests were two-sided. Continuous variables were compared between independent groups using the Kruskal–Wallis test.

To minimize the inflation of Type I error rates due to multiple comparisons, a Bonferroni correction was applied to the primary endpoints. The primary family of analyses comprised four planned comparisons: transfusion rate of any blood product, transfusion rate of RBC, number of RBC transfused per patient and the transfusion index. With four distinct comparisons in this primary analytical family (m = 4), the standard significance threshold of alpha = 0.05 was adjusted to a stricter alpha level of alpha= 0.0125 (0.05/4). Consequently, an individual comparison was considered statistically significant only if its calculated *p*-value was less than 0.0125. Secondary exploratory evaluations at individual time points between admission and discharge were interpreted descriptively to provide clinical context.

For all other outcomes and variables, statistical significance was defined as a two-sided α level of 0.05.

An exploratory multivariable logistic regression analysis was performed to investigate the association between ERAS–PBM implementation and RBC transfusion during hospitalization. Adjustment variables were selected based on clinical relevance, established transfusion risk factors, and data availability, while considering the number of outcome events to limit overfitting. Results are reported as adjusted odds ratios with 95% confidence intervals and interpreted as exploratory associations without causal inference. The analysis was restricted to patients with complete data for all variables included in the model.

Missing data were not imputed. Analyses were performed using the available data for each variable. Sample sizes are reported where they differ from the full cohort.

Statistical analysis was performed using JMP, version 7 (SAS Institute Inc., Cary, NC, USA, 1989–2023), and for the multivariable logistic regression analysis, Stata, version 19.0 (StataCorp LLC, College Station, TX, USA).

## 3. Results

### 3.1. ERAS Versus Pre-ERAS Group

Patient baseline characteristics (demographic and surgical data) for both groups are summarized in Table 1. Compared to the pre-ERAS group, the ERAS cohort included more patients with non-insulin-treated diabetes (7.4 vs. 12.7%) and a higher rate of isolated valve surgery (18.5% vs. 28.4%), but fewer cases of alcohol overconsumption (17.3% vs. 9.6%), peripheral arterial disease (25.9% vs. 10.0%), and cerebrovascular disease (10.5% vs. 4.4%). Cardiopulmonary bypass duration in minutes was also shorter in the ERAS group (94 [71, 125.5] vs. 70.5 [55, 90]). EuroSCORE II could be retrospectively calculated for 157 patients in the pre-ERAS cohort (96.9%) and 204 patients in the ERAS cohort (89.1%). The EuroSCORE II was also lower in the ERAS group (2.1% [1.2–4.3] vs. 1.3% [0.8–2.4]).

The perioperative transfusion outcomes are summarized in Table 2. Compared to the pre-ERAS group, the ERAS group received significantly fewer RBC units during hospitalization (0 [0, 2] vs. 0 [0, 0], *p* = 0.0005), and fewer patients required at least one RBC transfusion (38.9% vs. 23.6%, *p* = 0.0012). Overall transfusion rates (any blood product) were also lower (42.6% vs. 28.8%, *p* = 0.0049). Intraoperatively, fewer patients in the ERAS group received platelets (8.6% vs. 3.1%, *p* = 0.016), while FFP, RBC, transfusion of any blood product, and PCC did not meet the Bonferroni-corrected significance threshold. Fibrinogen was used more often in the ERAS group (24% vs. 41.9%, *p* = 0.0007). Transfusion rates within 12 h post-op were significantly lower in the ERAS group for RBCs (14.2% vs. 5.7%, *p* = 0.004) and all products combined (16.6% vs. 7.9%, *p* = 0.007), with no difference in platelets or FFP. Beyond 12 h post-op, RBC transfusion rates (29.6% vs. 17.5%, *p* = 0.004) and FFP use were significantly lower in the ERAS group.

Regarding *anemia* (Table 3), hemoglobin levels in g/L were higher in the ERAS group both at admission (134 [123, 144] vs. 139.5 [130, 147.7], *p* = 0.0009) and on postoperative day 5 (92 [87.2, 102.5] vs. 98 [90.5, 106.5], *p* = 0.015).

Other secondary outcomes (LOS and complications) are summarized in Table 4. Overall, postoperative complications were significantly less frequent in the ERAS group compared to the pre-ERAS group (66.7% vs. 46.3%, *p* < 0.001). MACE occurred more frequently in the pre-ERAS group (15.4% vs. 8.7%, *p* = 0.041), particularly heart failure (6.8% vs. 1.3%, *p* = 0.004). We found the same for renal failure (14.8% vs. 4.4%, *p* < 0.001). Pulmonary complications were also significantly less common in the ERAS group (28.4% vs. 13.1%, *p* < 0.001). The incidence of infections was reduced, although not significantly (21% vs. 14%, *p* = 0.069), while sepsis and septic shock occurred at similarly low rates in both groups. Regarding hospital stay, the LOS in nights was significantly shorter in the ERAS group compared to the pre-ERAS group (12 [8.7, 16] vs. 9 [7, 12], *p* < 0.001), as was mechanical ventilation time in hours (2.1 [0, 5.8] vs. 0 [0, 3], *p* < 0.001). The length of ICU stay in hours (25.9 [22.6, 50.3] vs. 26.5 [22.8, 46.8], *p* = 0.653) and ICU readmission rates (4.3% vs. 4.4%, *p* = 0.953) were similar between groups.

**Figure 2:**
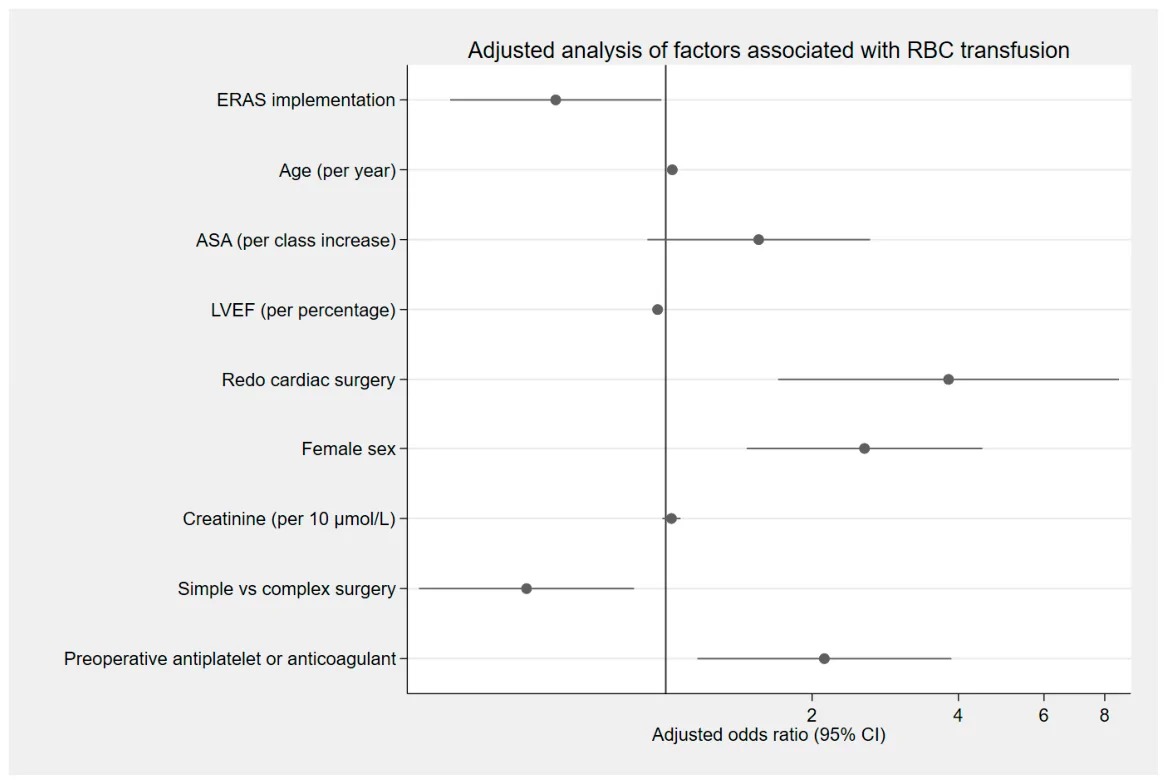
Exploratory multivariable analysis of factors associated with red blood cell transfusion during hospitalization. Points represent adjusted odds ratios and horizontal bars indicate 95% confidence intervals. The vertical line marks an odds ratio of 1. All displayed variables were included simultaneously in the logistic regression model (382 patients; 114 transfusion events). Odds ratios are expressed per year of age, one-class increase in ASA physical status, one percentage-point increase in LVEF, and 10 µmol/L increase in preoperative creatinine. Preoperative antithrombotic therapy denotes any antiplatelet or anticoagulant use. ASA: American Society of Anesthesiologists, ERAS: Enhanced Recovery After Surgery, LVEF: left ventricular ejection fraction, RBC: red blood cell.

An exploratory multivariable logistic regression analysis was performed to assess the association between ERAS–PBM implementation and RBC transfusion during hospitalization (Figure 2, Table S2 in Supplementary Materials). The analysis included 382 patients, of whom 114 received RBC transfusions. After adjustment for selected preoperative characteristics and surgical complexity, ERAS–PBM implementation remained associated with lower odds of RBC transfusion (adjusted OR 0.59, 95% CI 0.36–0.98, *p* = 0.041). Older age (OR 1.03 per year, *p* = 0.008), redo cardiac surgery (OR 3.82, *p* = 0.001), female sex (OR 2.56, *p* = 0.001), and preoperative antithrombotic or anticoagulant therapy (OR 2.12, *p* = 0.014) were associated with higher odds of RBC transfusion. Higher LVEF (OR 0.96 per percentage point, *p* = 0.003) and simple surgery (OR 0.52, *p* = 0.011) were associated with lower odds. ASA physical status and preoperative creatinine were not statistically significantly associated with RBC transfusion in this model. Simple surgery was defined as single-vessel coronary artery bypass grafting (CABG), single-valve surgery, or isolated ascending aortic replacement, whereas all other procedures were classified as complex surgery.

### 3.2. Anemia and Transfusion Related Results in the ERAS Group

**Figure 3:**
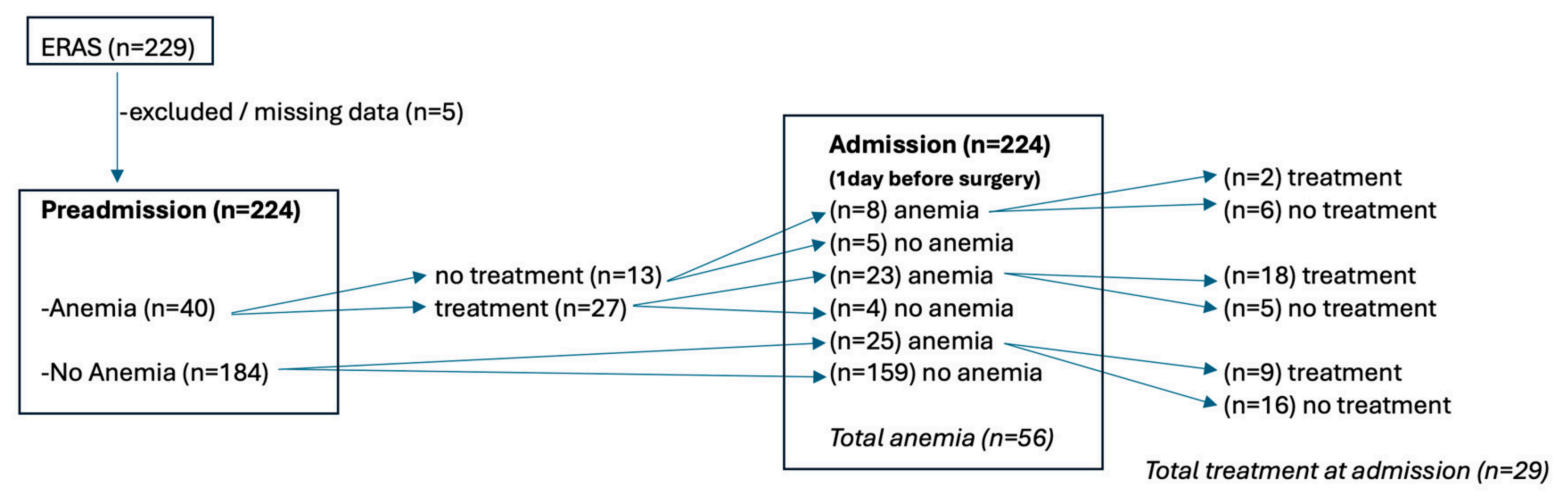
Patient classification according to anemia and treatment status.

The summary of characteristics related to the screening and treatment of anemia in patients from the ERAS group is visualized in Figure 3. In the ERAS group pre-admission Hb values were available for 224/229 patients. Pre-admission Hb was 143 g/L (IQR: 133, 150), with 17.8% presenting with anemia (40/224 patients). Of these anemic patients, 27/40 (67.5%) received treatment prior to admission. Admission Hb was available for 228/229 patients. Longitudinal analyses of anemia status from pre-admission to admission were restricted to patients with Hb measurements available at both time points.

At hospital admission, median Hb level was 139.5 g/L (IQR: 130, 147.7), and 56/228 patients (24.6%) were anemic. Among the 224 patients with both pre-admission and admission Hb measurements, 25 developed newly diagnosed anemia at admission.

We compared anemic and non-anemic patients within the ERAS group (Table 5). RBC transfusion rates during the hospital stay were significantly higher in patients anemic at admission compared to non-anemic patients (39.3% vs. 18.6%, *p* = 0.002). Renal failure was more frequent among anemic patients (10.7% vs. 2.3%, *p* = 0.02). No significant differences were observed in other complications or LOS.

Among the 65 patients who presented with anemia either at pre-admission or at hospital admission, an exploratory subgroup analysis compared those who received anemia treatment (*n* = 38) with those who did not (*n* = 27) (Table 6). The RBC transfusion rates during surgery (22.2% vs. 15.8%), in the first 12 postoperative hours (11.1% vs. 7.9%), and beyond 12 h (25.9% vs. 23.7%) did not show statistically significant differences between treated and untreated anemic patients. There was also no statistical difference in the secondary outcomes.

Also in the ERAS group, transfused patients (*n* = 54) had higher rates of MACE (20.4% vs. 5.2%, *p* = 0.002), renal failure (13.0% vs. 1.7%, *p* = 0.002), pulmonary complications (27.8% vs. 8.6%, *p* < 0.001) and infections (37% vs. 10.3%, *p* < 0.001) compared with non-transfused patients (*n* = 174) (Table 7). They also had longer mechanical ventilation times in hours (4 [0, 9] vs. 0 [0, 0.2]), ICU stay in hours (45.9 [26.6, 95.1] vs. 24.9 [22.4, 36.5]), and hospital stay in nights (12 [8, 15] vs. 8 [7, 11]) (all *p* < 0.001). Anemia at admission was more frequent among transfused patients (40.7% vs. 19.5%, *p* = 0.0016).

## 4. Discussion

In this study, we evaluated the impact of integrating a PBM program into our ERAS pathway. Implementation of this program was associated with lower allogeneic blood exposure, shorter length of stay, and fewer complications. Within the ERAS cohort, anemia at admission and exposure to allogeneic transfusions were associated with a higher incidence of selected postoperative complications.

Implementation of the integrated ERAS–PBM pathway was associated with a significant reduction in transfusion rates and RBC units administered, consistent with literature supporting restrictive transfusion strategies [1,5,12]. The transfusion index, representing the amount of blood products administered per transfused patient, did not differ significantly between groups. This suggests that the main difference was a reduction in the proportion of patients exposed to transfusion rather than in the amount administered once transfusion became necessary. In patients requiring transfusion, factors such as the magnitude of perioperative blood loss may become more important determinants of transfusion requirements than preoperative hemoglobin optimization.

In the exploratory multivariable logistic regression analysis, ERAS–PBM implementation remained associated with lower odds of RBC transfusion during hospitalization after adjustment for selected preoperative characteristics and surgical complexity (adjusted OR 0.59, 95% CI 0.36–0.98, *p* = 0.041). Although consistent with the unadjusted findings, the association was sensitive to covariate adjustment, and the confidence interval indicates uncertainty regarding its magnitude. These results should therefore be interpreted cautiously and do not establish a causal effect of the ERAS–PBM pathway.

Importantly, temporal changes in transfusion practice should also be considered when interpreting these findings. Reductions in RBC transfusion requirements over time have been reported in cardiac surgery cohorts [13]. However, temporal trends are not uniform across institutions; a recent nationwide study reported increasing RBC transfusion rates over time and substantial interhospital variability [14]. Therefore, although temporal changes in transfusion practices may have contributed to the observed differences, their respective contribution relative to the integrated ERAS–PBM pathway cannot be determined from our historical before–after design.

Within the ERAS group, patients presenting with anemia on the day of admission experienced significantly higher renal failure (10.7% vs. 2.3%) and were more likely to receive RBC transfusion during their hospital stay (39.3% vs. 18.6%) compared to non-anemic patients. These results are consistent with the existing literature describing preoperative anemia as a strong predictor of adverse outcomes in cardiac surgery [2,3,5,15]. Some experts have suggested that such integration (PBM and ERAS program) can enhance clinical outcomes by improving preoperative hemoglobin levels and minimizing transfusion needs [16,17]. Compared to other studies without a combined ERAS and PBM program, we observed higher hemoglobin levels at admission and lower transfusion rates. For example, data from 3500 consecutive cardiac surgery patients across seven academic hospitals showed a 26% prevalence of preoperative anemia (Hb < 12.5 g/dL; 22–30% across centers), and the hemoglobin concentration was 13.4 ± 1.7 g/dL [15].

These effects align with the pathway’s objectives, suggesting that multi-modal measures and real-time coordination (e.g., ERAS nurse, audit of adherence) are key drivers rather than any single measure. The ERAS and PBM concepts are based on the sum of marginal gains [18], in which many individual elements cannot be shown to make a statistically significant difference. Although multivariable analysis helps account for selected patient- and procedure-related differences, it cannot determine the contribution of individual components within the multimodal ERAS–PBM pathway. Recent work underscores the conceptual pairing of PBM with ERAS and provides practical order sets for PBM delivery in cardiac ERAS programs [16]. The measures proposed are in accordance with our program.

Our integration of the first pillar of PBM into the preoperative ERAS pathway has several strengths. The principal strength lies in the centralized coordination of the patient’s preoperative pathway by the ERAS nurse, which facilitates a coherent, standardized, and streamlined process. Furthermore, patients benefit from having a single, clearly identified point of contact, which enhances communication and continuity of care. Most importantly, the organization and follow-up of anemia management by the ERAS nurse facilitated coordination and delivery of the preoperative anemia pathway. The centralization of responsibilities within this role has also optimized interprofessional collaboration and reduced the administrative and clinical burden on cardiac surgeons and anesthesiologists, thereby promoting more efficient use of healthcare resources.

Considering our anemia treatment protocol, its design integrated several key reflections. Fundamentally, it was based on widely accepted clinical pathways and recommendations endorsed by relevant scientific societies [19,20]. Although the classification into four broad categories does not encompass all possible etiologies, it facilitates the timely management of the most treatable conditions within the limited preoperative timeframe. One important discussion point concerned the definition of iron deficiency, which varies considerably across the literature [21]. For the purposes of our algorithm, we adopted the criteria proposed in a modified Delphi study from Canada, which also serve to identify patients likely to benefit from intravenous (IV) iron therapy [22]. Furthermore, if iron, vitamin deficiencies, and renal anemia were excluded, anemia was considered non-specific and presumed to be related to an inflammatory condition [23]. In such cases, iv iron alone has no or only small effect and EPO-α therapy may be considered on an individual basis; however, it was not included in our outpatient anemia program, as this indication constitutes an off-label use in Switzerland and is not reimbursed in the outpatient setting. Due to this limitation, erythropoietin was administered at hospital admission. Despite being hospital-funded, this ultra-short-term treatment has proven effective [12] and is considered cost-beneficial under the DRG reimbursement system, as it reduces transfusion requirements, postoperative complications, and length of stay [24]. Addressing these implementation barriers at the level of the national healthcare system could reasonably augment hemoglobin gains and further decrease transfusion exposure.

An important finding from our cohort is the hemoglobin drop between pre-admission and admission among patients who were non-anemic at pre-admission but anemic at admission. The present data do not allow us to identify the causes of this decline, but as described already by Karski in 1999 [25], 37% of anemias were newly acquired in hospital, possibly due to blood drawings or preoperative coronary angiography for example. This needs further evaluation and possible interventions in the light of the second pillar of PBM, minimizing blood loss [6].

In particular, iron deficiency without anemia (IDWA) is increasingly recognized as clinically relevant in cardiac surgical patients and also associated with increased transfusion requirement and even mortality [26,27]. We considered including IV iron supplementation for patients with IDWA in our protocol; however, a recent meta-analysis on this topic concluded that scientific equipoise remains, underscoring the need for high-quality evidence before widespread implementation [28]. In our view, further research is required before integrating this approach into routine practice, as IV iron therapy carries a small but notable risk of allergic reactions [29], potential inconvenience for patients due to additional infusion visits, and added treatment costs.

### Limitations

This study’s single-center, historical before–after design inherently carries a risk of residual confounding. Although exploratory multivariable logistic regression adjusted for selected preoperative characteristics and surgical complexity, the historical before–after design cannot distinguish the effects of ERAS–PBM implementation from secular changes in clinical practice. No major changes in surgical techniques or perioperative practices unrelated to ERAS–PBM were identified between the study periods (2019 and 2023–2024), but more subtle changes cannot be excluded. Notably, fibrinogen administration differed between groups despite not being a specific target of the intervention. The ERAS cohort also had a lower EuroSCORE II, indicating a more favorable baseline surgical risk profile. Differences in patient characteristics, procedural mix, CPB duration, and perioperative coagulation management may therefore have contributed to the observed differences in transfusion rates despite adjustment. Model specification was explored using the available data, and the association between ERAS–PBM implementation and RBC transfusion was sensitive to covariate adjustment, with the confidence interval reflecting uncertainty in its magnitude. Furthermore, the logistic regression used a binary transfusion outcome and did not account for transfusion volume or timing, which were examined only in unadjusted analyses. Overall, these findings support an association between ERAS–PBM implementation and reduced RBC transfusion but do not establish causality.

Within the ERAS cohort, RBC transfusion was associated with a higher incidence of selected postoperative complications. However, this association cannot be interpreted as causal, as transfused patients may have experienced greater surgical complexity, more extensive bleeding, or greater perioperative clinical severity. This comparison is therefore subject to confounding by indication.

Hemoglobin levels on the day prior to surgery and on postoperative day 5 were significantly higher in the ERAS group. Whether this reflects the effectiveness of improved anemia management at admission remains uncertain, as hemoglobin data at pre-admission were not available for the pre-ERAS group. This gap limits our ability to determine whether the difference is attributable to the intervention or to baseline group differences. It limits direct comparison of hemoglobin trajectories between periods and introduces potential information bias. The fact that the patients’ general practitioners were involved from the start in our PBM educational program may have increased their awareness of the importance of anemia screening and treatment and may have influenced the results. Furthermore, the exploratory analysis of anemia treatment within the ERAS cohort showed no statistically significant differences, but its limited sample size precludes firm conclusions regarding treatment effectiveness, as a clinically relevant benefit cannot be excluded. Whatever the cause, higher perioperative hemoglobin levels may have contributed to the observed reduction in transfusion requirements.

Another important limitation relates to the evaluation of the anemia management pathway. Blood iron studies were not mandatory variables in the ERAS database, and ferritin values were frequently unavailable. Prospective and systematic collection of the laboratory variables required to characterize anemia etiology and assess adherence to the diagnostic and treatment algorithm should therefore be incorporated into future iterations of the program.

## 5. Conclusions

The combination of two structured and multimodal approaches— ERAS and PBM —within a single integrated program was associated with lower transfusion rates, fewer complications, and shorter length of stay in elective cardiac surgery. The novelty and strength of this work lie in the integrated implementation of ERAS–PBM as a single, multimodal program. Our findings add to the growing body of evidence highlighting the importance of anemia management and support the integration of ERAS and PBM strategies as a structured approach to optimize perioperative care. Further improvement of the pathway may include more systematic identification and treatment of IDWA and earlier use of erythropoietin in selected patients; these strategies require evaluation in future prospective studies.

## Figures and Tables

**Table 1:** Patient baseline and surgical characteristics.

	Pre-ERAS (*n* = 162)	ERAS (*n* = 229)
Men, *n* (%)	112 (69.1%)	173 (75.5%)
Women, *n* (%)	50 (30.9%)	56 (24.5%)
Age, median [IQR]	67 [59, 74]	63 [55, 71]
BMI, median [IQR]	25.7 [22.7, 29.7]	26.75 [23.9, 30.5]
Smoker, *n* (%)	39 (24.1%)	47 (20.5%)
Alcohol overconsumption, *n* (%)	28 (17.3%)	22 (9.6%)
Arterial hypertension treated, *n* (%)	110 (67.9%)	146 (63.7%)
Untreated arterial hypertension, *n* (%)	3 (1.8%)	9 (3.9%)
COPD, *n* (%)	25 (15.4%)	35 (15.3%)
Diabetic on medication without insulin, *n* (%)	12 (7.4%)	29 (12.7%)
Diabetic on insulin, *n* (%)	9 (5.6%)	12 (5.2%)
PAD, *n* (%)	42 (25.9%)	23 (10%)
Cerebrovascular disease, *n* (%)	17 (10.5%)	10 (4.4%)
CABG isolated, *n* (%)	47 (29%)	61 (26.6%)
Valve isolated, *n* (%)	30 (18.5%)	65 (28.4%)
CABG and valve, *n* (%)	19 (11.7%)	13 (5.7%)
Aorta, *n* (%)	11 (6.8%)	9 (3.9%)
Other isolated surgery, *n* (%)	30 (18.5%)	46 (20.1%)
CPB duration in minutes, median [IQR]	94 [71, 125.5]	70.5 [55, 90]
ASA status (*n*)	161	226
ASA2, *n* (%)	14 (8.7%)	27 (12.0%)
ASA3, *n* (%)	121 (75.2%)	160 (70.8%)
ASA4, *n* (%)	26 (16.1%)	39 (17.3%)
LVEF% before surgery, median [IQR]	60 [55, 65]	60 [55, 65]
Creatinine one day before surgery, median [IQR]	89.5 [75, 104]	89 [76, 102]
EuroSCORE II (%) **	2.1 [1.2, 4.3]	1.3 [0.8, 2.4]

Data presented as median with interquartile range (IQR), or parameter counts (n) with percentage (%). BMI: body mass index, COPD: chronic obstructive pulmonary disease, PAD: peripheral arterial disease, CABG: coronary artery bypass grafting, CPB: cardiopulmonary bypass, ASA: American Society of Anesthesiologists physical status classification, LVEF: left ventricular ejection fraction. ** EuroSCORE II was available for 157 pre-ERAS and 204 ERAS patients due to missing data.

**Table 2:** Perioperative transfusion outcomes.

	Pre–ERAS (*n* = 162)	ERAS (*n* = 229)	*p* Value
**Total during hospital stay**			
Transfusion (RBC, platelets 5Ui, FFP)	69 (42.6%)	66 (28.8%)	0.0049 **
RBC transfusion	63 (38.9%)	54 (23.6%)	0.0012 **
Nbr RBC per patient	0 [0, 2]	0 [0, 0]	0.0005 **
Transfusion index: nbr RBC/transfused patient	2 [1, 4]	2 [1, 3]	0.167
**During surgery**			
Transfusion (RBC, platelets 5Ui, FFP)	39 (24.1%)	36 (15.7%)	0.039
RBC transfusion	27 (16.7%)	22 (9.6%)	0.039
Nbr RBC per patient	0 [0, 0]	0 [0, 0]	0.038
Platelets 5Ui transfusion	14 (8.6%)	7 (3.1%)	0.016
Nbr Platelets 5Ui per patient	0 [0, 0]	0 [0, 0]	0.018
FFP transfusion	20 (12.3%)	29 (12.7%)	0.925
Nbr FFP per patient	0 [0, 0]	0 [0, 0]	0.988
PCC ***	6 (3.7%)	5 (2.2%)	0.536
Fibrinogen	39 (24.1%)	96 (41.9%)	<0.001
**In the first 12 h after surgery**			
Transfusion (RBC, platelets 5Ui, FFP)	27 (16.6%)	18 (7.9%)	0.007
RBC transfusion	23 (14.2%)	13 (5.7%)	0.004
Nbr RBC per patient	0 [0, 0]	0 [0, 0]	0.005
Platelets 5Ui transfusion	2 (1.2%)	3 (1.3%)	1.000
Platelets 5Ui per patient	0 [0, 0]	0 [0, 0]	0.949
FFP transfusion	13 (8%)	14 (6.1%)	0.462
Nbr FFP per patient	0 [0, 0]	0 [0, 0]	0.461
**More than 12 h after surgery**			
Transfusion (RBC, platelets 5Ui, FFP)	49 (30.2%)	40 (17.5%)	0.003
RBC transfusion	48 (29.6%)	40 (17.5%)	0.004
Nbr RBC transfusion per patient	0 [0, 1]	0 [0, 0]	0.005
Platelets 5Ui transfusion	3 (1.9%)	0 (0%)	0.070
Nbr Platelets 5Ui per patient	0 [0, 0]	0 [0, 0]	0.039
FFP transfusion	4 (2.5%)	0 (0%)	0.029
Nbr FFP per patient	0 [0, 0]	0 [0, 0]	0.039

Data presented as *n* (%) for categorical variables and as median with interquartile range (IQR). RBC: red blood cell, FFP: fresh frozen plasma, Ui: unit, Nbr: number, PCC: prothrombin complex concentrate. ** Statistically significant after Bonferroni correction. *** PCC during surgery: Data were missing for 2 patients in the ERAS group and 1 patient in the pre-ERAS group (*n* = 388).

**Table 3:** Anemia prevalence and perioperative hemoglobin levels.

	Pre-ERAS *n* = 161	ERAS *n* = 228	*p* Value
Anemia at admission	68 (42.2%)	56 (24.6%)	0.0002
Hb value at admission	134 [123, 144]	139.5 [130, 147.7]	0.0009
Hb value at day 5	92 [87.2, 102.5]	98 [90.5, 106.5]	0.015
Delta Hb (day 5—operation day)	−34 [−44, −26]	−38 [−46, −28]	0.305

Data presented as *n* (%) for categorical variables, or as median with interquartile range (IQR). Hb: hemoglobin value (g/L). One missing data point for Hb value at admission in the pre-ERAS group. One missing data point for Hb value at admission in the ERAS group.

**Table 4:** Clinical outcomes in the pre-ERAS and ERAS groups.

	Pre-ERAS (*n* = 162)	ERAS (*n* = 229)	Total (*n* = 391)	*p* Value
LOS (nights)	12 [8.7, 16]	9 [7, 12]	10 [8, 14]	<0.001
ICU stay (hours)	25.9 [22.6, 50.3]	26.5 [22.8, 46.8]	26.2 [22.7, 48.5]	0.653
ICU readmission **	7 (4.3%)	10 (4.4%)	17 (4.4%)	0.953
Any listed complications	108 (66.7%)	106 (46.3%)	214 (54.7%)	<0.001
Free from any listed complications	54 (33.3%)	123 (53.7%)	177 (45.3%)	<0.001
MACE	25(15.4%)	20(8.7%)	45(11.5%)	0.041
Stroke	4 (2.5%)	9 (3.9%)	13 (3.3%)	0.427
Heart failure	11 (6.8%)	3 (1.3%)	14 (3.6%)	0.004
Cardiogenic shock	14 (8.6%)	9 (3.9%)	23 (5.9%)	0.051
Myocardial infarction	1 (0.6%)	1 (0.4%)	2 (0.5%)	1.000
30-day death	2 (1.2%)	2 (0.9%)	4 (1.0%)	1.000
Renal failure	24 (14.8%)	10 (4.4%)	34 (8.7%)	<0.001
Renal replacement therapy	1 (0.6%)	3 (1.3%)	4 (1%)	0.645
Pulmonary complication	46 (28.4%)	30 (13.1%)	76 (19.4%)	<0.001
Thrombotic (non-stroke)	4 (2.5%)	1 (0.4%)	5 (1.3%)	0.165
Infection	34 (21%)	32 (14%)	66 (16.9%)	0.069
Sepsis	2 (1.2%)	2 (0.9%)	4 (1%)	1.000
Septic shock	2 (1.2%)	2 (0.9%)	4 (1%)	1.000
Mechanical ventilation time (hours)	2.1 [0, 5.8]	0 [0, 3]	0 [0, 4.5]	<0.001

Data presented as *n* (%) for categorical variables, or as median with interquartile range (IQR). LOS: length of stay, ICU: intensive care unit. Definitions of postoperative complications and clinical outcomes are provided in Supplementary Materials, Table S1. ** ICU readmission data were unavailable for four patients in the ERAS group (*n* = 225 for this outcome).

**Table 5:** Clinical outcomes according to anemia status at hospital admission in the ERAS group.

	Anemia at Admission *n* = 56	No anemia at Admission *n* = 172	*p* Value
RBC transfusion at all during the stay	22 (39.3%)	32 (18.6%)	0.002
Any listed complication	32 (57.1%)	73 (42.4%)	0.06
Major adverse cardiovascular events	6 (10.7%)	14 (8.1%)	0.59
Renal failure	6 (10.7%)	4 (2.3%)	0.02
Renal replacement therapy	2 (3.6%)	1 (0.6%)	0.15
Pulmonary complication	9 (16.1%)	21 (12.2%)	0.46
Thrombotic (non-stroke)	0 (0%)	1 (0.6%)	1.00
Infection	14(25%)	24 (14%)	0.054
Sepsis	0 (0%)	2 (1.2%)	1.00
Septic shock	0 (0%)	2 (1.2%)	1.00
Mechanical ventilation time (hours)	0 [0, 3]	0 [0, 3]	0.98
ICU duration (hours)	27.1 [23.4, 48.2]	26.4 [22.6, 45.7]	0.41
LOS (nights)	10 [8, 13]	8 [7, 12]	0.08

Categorical variables are presented as *n* (%). Continuous variables are presented as median with interquartile range (IQR). RBC: red blood cell, LOS: length of stay, ICU: intensive care unit. One patient was excluded because of missing admission hemoglobin data.

**Table 6:** Clinical outcomes according to anemia treatment status in the ERAS group.

	No Treatment *n* = 27	Treatment *n* = 38	*p* Value
Hb value on day 5	92.5 [85, 102.5]	95 [87.5, 96.5]	0.98
RBC transfusion during surgery	6 (22.2%)	6 (15.8%)	0.53
nbr RBC	0 [0, 0]	0 [0, 0]	0.42
RBC transfusion during first 12 h	3 (11.1%)	3 (7.9%)	0.67
nbr RBC	0 [0, 0]	0 [0, 0]	0.69
RBC transfusion 12 h after surgery	7 (25.9%)	9 (23.7%)	0.84
nbr RBC 12 h	0 [0, 1]	0 [0, 0.2]	0.82
RBC transfusion overall during the stay	10 (37%)	14 (36.8%)	0.99
Any listed complications	17 (63%)	22 (57.9%)	0.68
Major adverse cardiovascular events	4 (14.8%)	3 (7.9%)	0.44
Renal failure	2 (7.4%)	4 (10.5%)	1.00
Renal replacement therapy	0	2 (5.3%)	0.51
Pulmonary complication	5 (18.5%)	8 (21.1%)	0.80
Stroke	1 (3.7%)	1 (2.6%)	1.00
Infection	5 (18.5%)	10 (26.3%)	0.46
Sepsis	1(3.7%)	0	0.42
Septic shock	0	1(2.6%)	1.00
Mechanical ventilation time (hours)	0 [0, 4.5]	0 [0, 4.1]	0.60
ICU duration (hours)	29 [23.4, 75.8]	26.1 [23, 46.5]	0.38
LOS (nights)	10 [8, 13]	10 [8, 15]	0.65

Categorical variables are presented as *n* (%). Continuous variables are presented as median with interquartile range (IQR). RBC: red blood cell, LOS: length of stay, ICU: intensive care unit.

**Table 7:** Clinical outcomes according to transfusion status in the ERAS group.

	Transfused *n* = 54	Not Transfused *n* = 174	*p* Value
Admission—Hb value (g/L)	134 [122.7, 141.5]	142 [131.7, 148]	0.0016
Anemia at admission	22 (40.7%)	34 (19.5%)	0.0016
Any listed complication (*n*, %)	31 (57.4%)	74 (42.5%)	0.055
MACE	11 (20.4%)	9 (5.2%)	0.002
Renal failure	7 (13%)	3 (1.7%)	0.002
Renal replacement therapy	2 (3.7%)	1 (0.6%)	0.140
Pulmonary complication	15 (27.8%)	15 (8.6%)	<0.001
Thrombotic (non-stroke)	1 (1.9%)	0 (0%)	0.237
Infection	20 (37%)	18 (10.3%)	<0.001
Sepsis	2 (3.7%)	0 (0%)	0.055
Septic shock	1(1.9%)	1 (0.6%)	0.418
Mechanical ventilation time (hours)	4 [0, 9]	0 [0, 0.2]	<0.001
ICU duration (hours)	45.9 [26.6, 95.1]	24.9 [22.4, 36.5]	<0.001
LOS (nights)	12 [8, 15]	8 [7, 11]	<0.001

Categorical variables are presented as *n* (%). Continuous variables are presented as median with interquartile range (IQR). MACE: major cardiovascular event, LOS: length of stay, ICU: intensive care unit. Analyses were restricted to 228 patients (54 transfused and 174 non-transfused), excluding one patient with missing admission hemoglobin data.

## Data Availability

The datasets used and/or analyzed during the current study are available from the corresponding authors on reasonable request.

## Supplementary Materials

The following supporting information can be downloaded at https://www.mdpi.com/article/10.3390/jcm15218447/s1: Table S1: Definitions of postoperative complications and clinical outcomes; Table S2: Exploratory multivariable logistic regression analysis of factors associated with RBC transfusion during hospitalization.

## Abbreviations

The following abbreviations are used in this manuscript:

CHUV	Lausanne University Hospital
UNIL	Université de Lausanne
ERAS	Enhanced recovery after surgery
PBM	Patient blood management
LOS	Length of stay
ICU	Intensive care unit
REDCap	Research Electronic Data Capture
RBC	Red blood cell
FFP	Fresh frozen plasma
PCC	Prothrombin complex concentrate
MACE	Major adverse cardiovascular events
Hb	Hemoglobin
TSAT	Transferrin saturation
Creat	Creatinine
MCV	Mean corpuscular volume
eGFR	Estimated glomerular filtration rate
EPOα	Erythropoietin alpha
ASA	American Society of Anesthesiologists
LVEF	Left ventricular ejection fraction
BMI	Body mass index
CPB	Cardiopulmonary bypass
IQR	Interquartile range
COPD	Chronic obstructive pulmonary disease
PAD	Peripheral arterial disease
CABG	Coronary artery bypass grafting
SD	Standard deviation
Nbr	Number
DRG	Diagnosis-Related Group
IDWA	Iron deficiency without anemia
CER-VD	Commission cantonale d’éthique de la recherche sur l’être humain du Canton de Vaud
