# Supplementary material for: An Integrated ERAS–PBM Pathway for Cardiac Surgery: Insights and Outcomes
Source: J Clin Med. 2026 Oct 30;15(21):8447. doi: 10.3390/jcm15218447 (PMC13763802; doi:10.3390/jcm15218447)
Supplement: Supplementary file 1 [file jcm-15-08447-s001.zip › jcm-4543443-supplementary.pdf]

Supplementary Materials

Table S1. Definitions of postoperative complications and clinical outcomes

Outcome category	Definition used in the study
MACE	Myocardial infarction, stroke, acute heart failure, cardiac arrest, or death
Pulmonary complications	Pneumonia, pleural effusion, respiratory failure, or pneumothorax
Infectious complications	Pneumonia, wound infection, urinary tract infection, mediastinitis, sternotomy infection, vascular access or vein graft infection, sepsis, or septic shock
Renal complications	Renal failure or need for transient renal replacement therapy
Thrombotic events	Pulmonary embolism or deep venous thrombosis

*MACE: major adverse cardiovascular events.

Table S2. Exploratory multivariable logistic regression analysis of factors associated with RBC transfusion during hospitalization

Variable	Adjusted OR	95% CI	p-value
ERAS implementation	0.59	0.36–0.98	0.041
Age, per year	1.03	1.01–1.06	0.008
ASA physical status, per class increase	1.55	0.92–2.63	0.102
LVEF, per percentage point	0.96	0.94–0.99	0.003
Redo cardiac surgery	3.82	1.70–8.56	0.001
Female sex	2.56	1.47–4.48	0.001
Preoperative creatinine, per 10 μmol/L	1.03	0.98–1.07	0.217
Simple versus complex surgery	0.52	0.31–0.86	0.011
Preoperative antiplatelet or anticoagulant therapy	2.12	1.16–3.87	0.014

Values are adjusted odds ratios (ORs) with 95% confidence intervals (CIs). All listed variables were included simultaneously in the model, which included 382 patients and 114 transfusion events. ASA: American Society of Anesthesiologists, ERAS: Enhanced Recovery After Surgery, LVEF: left ventricular ejection fraction, RBC: red blood cell.
